# Acute Encephalopathy during First Pembrolizumab Infusion Followed by Delayed Multisystem Immune Toxicity in Recurrent Vaginal Cancer: A Case Report

**DOI:** 10.1159/cro/abzag026

**Published:** 2026-08-28

**Authors:** Caleigh E. Smith, Laura E. Dussan, Johanna A. Kelley, Lindsey Beffa

**Affiliations:** a Obstetrics and Gynecology Institute, Cleveland Clinic, Cleveland, OH, USA; b Digestive Disease and Surgery Institute, Cleveland Clinic, Cleveland, OH, USA

**Keywords:** Vaginal cancer, Immune checkpoint inhibitor, Immunotherapy, Encephalopathy, Immune-related adverse events

## Abstract

**Introduction:**

Primary vaginal cancer is a rare malignancy, and treatment strategies are often extrapolated from cervical cancer due to limited prospective data. Immune checkpoint inhibitors (ICIs), including pembrolizumab, have demonstrated efficacy in gynecologic cancers but are associated with immune-related adverse events (irAEs), which can involve multiple organ systems. Neurologic irAEs are uncommon but may be severe and diagnostically challenging.

**Case Presentation:**

We present a 67-year-old woman with recurrent HPV-associated squamous cell carcinoma of the vagina who developed acute encephalopathy after receiving bevacizumab and paclitaxel and within minutes of initiating pembrolizumab during her first treatment cycle. Symptoms included confusion, hallucinations, and agitation. Extensive evaluation did not identify an infectious, metabolic, structural, epileptic, malignant, or antibody-mediated cause. She required intensive care support, and her mental status improved with supportive management. Although the temporal association raised concern for an immune-mediated neurologic event, the immediate onset, exposure to multiple agents, absence of objective inflammatory findings, and improvement without corticosteroids limited causal attribution. Four weeks later, she developed suspected multisystem irAEs characterized by myositis, transaminitis, and worsening thyroid dysfunction, necessitating high-dose corticosteroids and multidisciplinary management. Pembrolizumab was discontinued, and she was transitioned to single-agent chemotherapy.

**Conclusion:**

This case highlights the diagnostic complexity of acute neurologic symptoms arising during ICI-containing combination therapy, followed by delayed multisystem immune-related toxicity. Clinicians should recognize that acute neurologic symptoms may arise during the first immunotherapy treatment and require prompt evaluation for immune-mediated toxicity as well as infusion-related, medication-associated, vascular, infectious, metabolic, and malignant causes. Early recognition and multidisciplinary collaboration are critical to reducing morbidity. Given the limited data on immunotherapy in vaginal cancer, continued reporting of such cases is essential to inform safety, guide management, and improve patient selection in this rare disease.

## Plain Language Summary

Vaginal cancer is rare, and treatment decisions are often based on research from similar cancers, such as cervical cancer. Newer treatments called immune checkpoint inhibitors are increasingly used for advanced cancers. These medications help the immune system recognize and attack cancer cells, but they can also cause the immune system to damage normal tissues. These complications are called immune-related adverse events. We describe a patient with recurrent vaginal cancer who developed sudden confusion, agitation, and hallucinations during her first treatment cycle. She had already received bevacizumab and paclitaxel, and her symptoms began within minutes of starting pembrolizumab, an immune checkpoint inhibitor. Extensive testing did not identify a clear infectious, metabolic, structural, seizure-related, or cancer-related cause. Her symptoms gradually improved with supportive care and without steroid treatment. Four weeks later, she developed suspected immune-related side effects involving her muscles, liver, and thyroid gland. These complications required high-dose steroids and care from several medical specialists. Pembrolizumab was stopped, and her cancer treatment was changed. This case highlights the difficulty of determining the cause of sudden neurologic symptoms during combination cancer treatment. Such symptoms may be related to immunotherapy, another medication, or an infusion-related reaction. Prompt evaluation and collaboration among specialists are important to reduce harm and guide future treatment decisions.

## Introduction

Primary vaginal cancer is an uncommon malignancy, accounting for approximately 1–2% of all gynecologic cancers [1]. Squamous cell carcinoma (SCC) is the predominant histology, while adenocarcinoma, melanoma, and sarcoma account for less common subtypes [2]. Established risk factors overlap with those for cervical cancer and include smoking, high-grade intraepithelial lesions, immunosuppression, and persistent human papillomavirus infection [3]. Because of its rarity, treatment recommendations are based largely on retrospective studies and extrapolation from cervical cancer rather than prospective vaginal cancer-specific trials [1, 3].

Immune checkpoint inhibitors (ICIs) have emerged as an important component of treatment for several advanced gynecologic malignancies. By inhibiting immune regulatory pathways such as programmed cell death protein 1, programmed death-ligand 1, and cytotoxic T-lymphocyte-associated protein 4, these agents can enhance anti-tumor immunity and achieve durable clinical responses in select patients [4]. However, they are also associated with a distinct spectrum of immune-related adverse events (irAEs) that can involve nearly any organ system and range from mild to life-threatening [4–6]. Neurologic irAEs are particularly rare but clinically significant because their manifestations are often nonspecific, diagnostic testing may be unrevealing, and delayed recognition can result in substantial morbidity [7].

Reports of neurologic irAEs in gynecologic malignancies remain limited, particularly in vaginal cancer. We present a patient with recurrent vaginal SCC who developed acute encephalopathy during the first pembrolizumab-containing treatment cycle, followed 4 weeks later by suspected multisystem immune-related toxicity. This case highlights the diagnostic uncertainty surrounding acute neurologic symptoms during combination systemic therapy, the importance of considering competing treatment-related and non-treatment-related etiologies, and the need to distinguish temporal association from definitive immune-mediated causality. This case report was prepared in accordance with the CARE guidelines, and the completed CARE checklist is provided as online supplementary material (for all online suppl. material, see https://doi.org/10.1159/cro/abzag026).

## Case Presentation

The patient is a 67-year-old woman with past medical history of type 2 diabetes, hypertension, hypothyroidism, stage 3a chronic kidney disease, and supraventricular tachycardia. She had previously undergone a hysterectomy with bilateral salpingo-oophorectomy for abnormal uterine bleeding. Final pathology was benign. Years later, she was diagnosed with an HPV-associated stage III SCC of the vagina. She received chemoradiation with weekly cisplatin 40 mg/m^2^ as a radiosensitizer, with intravenous hydration and close renal monitoring during treatment, followed by interstitial brachytherapy. The tumor was mismatch repair (MMR) proficient with a programmed death-ligand 1 (PD-L1) combined positive score (CPS) of 5. The patient’s overall clinical course is summarized in Table 1.

**Table 1.** Clinical timeline of acute encephalopathy and delayed multisystem immune-related toxicity following pembrolizumab-containing therapy

Time point	Clinical event	Key findings	Management/outcome
Initial diagnosis	Stage III vaginal SCC	HPV positive; MMR proficient; PD-L1 CPS 5	Chemoradiation with cisplatin, EBRT, and interstitial brachytherapy
4 month post-treatment	Recurrent metastatic disease	New pulmonary nodules; lung biopsy confirmed metastatic SCC of vaginal origin	Planned carboplatin, paclitaxel, pembrolizumab, and bevacizumab
Day 0	First systemic therapy infusion	Bevacizumab and paclitaxel tolerated; acute encephalopathy within minutes of pembrolizumab initiation (confusion, agitation, hallucinations)	Carboplatin withheld; transferred to emergency department
Days 0–12	First hospitalization	Neuroimaging (CT, MRI), EEG, CSF analysis, infectious workup, and metabolic evaluation unrevealing	ICU admission; supportive care; mental status improved; pembrolizumab discontinued
Weeks 4–5	Readmission with delayed multisystem irAEs	Nausea, transaminitis, worsening thyroid function, elevated CK	Clinically diagnosed with suspected delayed multisystem ICI-related toxicity; treated with high-dose corticosteroids
2-month follow-up	Clinical recovery	Clinically stable	Received single-agent carboplatin, tolerated well

HPV, human papilloma virus; SCC, squamous cell carcinoma; MMR, mismatch repair; PD-L1, programmed death-ligand 1; CPS, combined positive score; EBRT, external beam radiation therapy; EEG, electroencephalography; CSF, cerebrospinal fluid; CK, creatine kinase; irAEs, immune-related adverse events; ICI, immune checkpoint inhibitor.

Four months following definitive chemoradiation, surveillance imaging demonstrated new bilateral pulmonary nodules. CT-guided lung biopsy confirmed metastatic SCC of vaginal origin. Systemic therapy with carboplatin, paclitaxel, pembrolizumab, and bevacizumab was planned for treatment of her recurrent metastatic disease. Pembrolizumab use was supported by PD-L1 positivity and extrapolation from cervical cancer data in the absence of vaginal cancer-specific trials. At initiation of systemic therapy, the patient received standard premedication followed by bevacizumab, paclitaxel, and pembrolizumab. After receiving bevacizumab and paclitaxel, she reported hand numbness and fatigue but remained stable. Within minutes of initiating pembrolizumab, she developed confusion, agitation, and hallucinations. The planned carboplatin was withheld, and she was transferred to the emergency department for evaluation.

On examination, the patient was disoriented with impaired attention and limited eye contact. The neurologic exam was otherwise normal, without focal neurologic deficits. Initial laboratory studies in the emergency department, including complete blood count, electrolytes, thyroid function, ammonia, and urinalysis, did not identify a clear metabolic cause. Initial neuroimaging included a noncontrast computed tomography (CT) brain attack protocol and CT angiography of the head and neck with intravenous contrast, neither of which demonstrated an acute intracranial abnormality, large-vessel occlusion, or other vascular cause of her symptoms. CT of the chest, abdomen, and pelvis likewise showed no acute abnormalities. Empiric antibiotics were initiated while urine and blood cultures were pending. Benzodiazepines provided only transient improvement in mental status, and her encephalopathy persisted, so she was transferred to a tertiary care center and admitted directly to the intensive care unit (ICU).

Neurology and psychiatry were consulted. The differential diagnosis included medication-induced or infusion-related encephalopathy, an immune-related adverse event, infectious or autoimmune encephalitis, seizure, vascular pathology, and leptomeningeal carcinomatosis. Brain magnetic resonance imaging (MRI) showed no acute intracranial abnormality, and a 20-min electroencephalogram (EEG) showed no seizure activity. Because she remained encephalopathic approximately 48 h after presentation, lumbar puncture (LP) was pursued. An initial bedside attempt was unsuccessful, followed by successful fluoroscopy-guided LP at L4–L5 by interventional radiology, yielding clear, colorless cerebrospinal fluid (CSF).

CSF contained 0 red blood cells and 2 total nucleated cells/µL (reference range, 0–5 cells/µL), with a manual differential of 93% lymphocytes and 7% monocytes. Protein was 39 mg/dL (15–45 mg/dL), and glucose was 82 mg/dL (40–70 mg/dL), compared with a serum glucose of 170 mg/dL obtained within 1 h. Gram stain and bacterial culture showed no growth, and a multiplex meningitis/encephalitis polymerase chain reaction panel was negative for the tested bacterial, viral, and fungal pathogens. Cytology showed no malignant cells, and autoimmune encephalopathy testing detected no informative autoantibodies, although this did not exclude autoimmune or paraneoplastic encephalopathy. Opening pressure and CSF lactate were not documented.

With continued supportive care, including sedation and antipsychotic therapy, her mental status gradually improved over several days. Antibiotics were discontinued following finalized negative urine, blood, and CSF cultures. Although no infectious, structural, epileptic, metabolic, malignant, or antibody-mediated etiology was identified, the absence of inflammatory CSF findings and spontaneous improvement without corticosteroids limited diagnostic certainty. Given the close temporal association with treatment, medication-induced encephalopathy, an acute infusion-related reaction, and possible immune-mediated encephalopathy remained leading considerations.

Her course was further complicated by severe vulvar pain with new blistering lesions, also thought to be related to treatment. No additional immune-related toxicities were identified during this admission. She was discharged on hospital day 12 with a symptom-directed regimen. No further pembrolizumab was administered following this event.

Four weeks later, she was re-admitted with refractory nausea and elevated aspartate aminotransferase (AST), 144 U/L (reference range, 13–35 U/L); alanine aminotransferase (ALT), 82 U/L (7–38 U/L); thyroid-stimulating hormone (TSH), 61 mIU/L (0.27–4.20 mIU/L); and creatine kinase (CK), 1,444 U/L (42–196 U/L). Additional evaluation, including complete blood count, adrenocorticotropic hormone, cortisol, hepatitis panel, and right upper quadrant ultrasound, was unrevealing. Cardiac evaluation was notable for a high-sensitivity troponin T of 454 ng/L (<12 ng/L) and N-terminal pro-B-type natriuretic peptide of 1,073 pg/mL (<125 pg/mL). Cardiac MRI and echocardiogram were normal, suggesting demand ischemia rather than myocarditis. She was clinically diagnosed with suspected delayed multisystem immune-related toxicity characterized by myositis, transaminitis, and worsening thyroid function, after alternative causes were not identified. No organ biopsy was performed.

These toxicities were managed via multidisciplinary collaboration with rheumatology, endocrinology, and cardiology. Methylprednisolone was started immediately on admission at 2 mg/kg/day per National Comprehensive Cancer Network (NCCN) guidelines for suspected high-grade immune-mediated toxicities [8] and increased to 2.5 mg/kg/day for 3 days due to inadequate clinical response. A prolonged steroid taper was initiated after conclusion of high-dose IV corticosteroids, and she received *Pneumocystis jirovecii* pneumonia prophylaxis with inhaled pentamidine given the anticipated duration of immunosuppression. Endocrinology managed worsening hypothyroidism in the setting of pre-existing Hashimoto thyroiditis, as well as pre-existing diabetes complicated by steroid-induced hyperglycemia. Her home levothyroxine dose was increased from 125 to 175 µg daily because of markedly elevated TSH and low thyroid hormone levels. Thyroid-stimulating immunoglobulin was negative, and thyroid peroxidase antibodies were positive, consistent with underlying Hashimoto thyroiditis. Repeat testing prior to discharge showed improvement in TSH and normalization of thyroxine on this new dose. She was discharged on levothyroxine 175 µg daily with repeat thyroid studies planned in 2 weeks and outpatient endocrinology follow-up. She was discharged on hospital day 6 with plans for close outpatient monitoring including remote follow-up that week and serial laboratory studies. At 2-month follow-up, she remained clinically stable and received her first cycle of single-agent carboplatin without complication.

## Discussion

This case highlights the diagnostic complexity of acute neurologic symptoms occurring during an immune checkpoint inhibitor-containing combination regimen and the challenge of distinguishing a neurologic immune-related adverse event from other treatment-associated processes. Primary vaginal cancer is rare, and treatment strategies are frequently adapted from cervical cancer because prospective trials are lacking [1, 2]. Evidence for the efficacy of immunotherapy regimens for vaginal or vulvar cancer is limited to basket trials, case series, and case reports with small sample sizes [9, 10]. In contrast, large, randomized trials in advanced endometrial and cervical cancers demonstrate durable responses to pembrolizumab combined with chemotherapy [11–14], and these data are often extrapolated to vaginal SCC because of histologic and molecular overlap between cervical and vaginal cancers. As a result, patients with vaginal cancer are increasingly exposed to immune checkpoint inhibitors, making recognition of associated toxicities clinically important.

ICIs are associated with irAEs, which can impact multiple organ systems and range from mild to life-threatening [4, 6]. Neurologic irAEs in particular are associated with significant morbidity and mortality, though they remain rare and are less frequently reported in the literature [7]. These toxicities encompass a broad spectrum of peripheral and central nervous system (CNS) manifestations, including encephalitis, aseptic meningitis, peripheral neuropathies, and myasthenia gravis [15]. The overall incidence of neurologic irAEs is less than 5%, with encephalopathy, seizures, and focal neurologic deficits being the most commonly reported CNS toxicities [6]. Proposed mechanisms include immune-mediated inflammation and cross-reactivity against neural antigens, though the precise pathophysiology remains incompletely understood [15].

The onset of neurologic irAEs is highly variable, with some cases occurring even after treatment discontinuation [15]. In a recent institutional cohort and systematic review of ICI-associated encephalitis, symptom onset ranged from less than 24 h to more than 1 year after treatment initiation, with a median onset of 62 days [16]. Individual early-onset reports include ICI-associated autoimmune encephalitis presenting with expressive aphasia 3 days after the first pembrolizumab-containing treatment cycle [17]. Thus, neurologic immune toxicity may occur early, but onset during the infusion itself remains exceptionally unusual. In the present case, this extreme temporal proximity raises the possibility of an acute infusion-related, cytokine-mediated, or medication-associated process rather than classic delayed autoimmune encephalitis.

The extensive neurologic evaluation in this case illustrates both the breadth of the differential diagnosis and the limitations of current diagnostic testing. Neuroimaging showed no acute structural or vascular abnormality, and EEG showed no seizure activity. CSF showed no pleocytosis or elevated protein, with glucose appropriate relative to concurrent serum glucose, negative bacterial culture and multiplex infectious testing, negative cytology, and no informative autoimmune encephalopathy antibodies. These findings reduced concern for infectious meningitis or encephalitis, seizure, leptomeningeal carcinomatosis, and several antibody-associated autoimmune or paraneoplastic syndromes. However, normal imaging, EEG, CSF findings, and antibody testing do not definitively exclude an immune-mediated neurologic adverse event [4, 7]. The absence of objective central nervous system inflammation and the patient’s gradual improvement with supportive care alone, without corticosteroids, nevertheless limited diagnostic certainty.

Causal attribution was further complicated by administration of multiple systemic agents in close succession. The patient received bevacizumab and paclitaxel before pembrolizumab, while carboplatin was withheld after symptom onset. Although rare, acute transient encephalopathy has been reported within hours of paclitaxel infusion, including presentations with confusion and hallucinations [18]. Bevacizumab has also been associated with posterior reversible encephalopathy syndrome (PRES), which may present with altered mental status, seizures, headache, or visual symptoms [19]. The absence of characteristic MRI findings made PRES less likely in this case. Accordingly, the initial event is best characterized as acute encephalopathy of uncertain etiology, with medication-induced, infusion-related, cytokine-mediated, and possible immune-mediated mechanisms remaining plausible.

Four weeks later, the development of myositis and transaminitis, accompanied by worsening thyroid dysfunction, was more consistent with delayed multisystem immune toxicity and required high-dose corticosteroids. These subsequent toxicities support the possibility that the initial event occurred within a broader spectrum of pembrolizumab-associated toxicity, but they do not establish that the acute encephalopathy was definitively immune mediated.

Data specific to irAEs in gynecologic malignancies remain limited, with most available reports derived from broader oncology populations such as melanoma, lung, and breast cancers [15]. There are infrequent reports of neurologic irAEs in cervical cancer cohorts, with one case of immune-related encephalitis reported in the pembrolizumab arm of the KEYNOTE-826 trial, which ultimately resulted in a participant death [11]. To our knowledge, no prior published cases have described neurologic irAEs in patients with vaginal cancer receiving pembrolizumab. However, the current report is limited by its single-patient design and inability to establish causality beyond temporal association.

In conclusion, acute neurologic symptoms can occur during the first ICI-containing infusion, but temporal association alone may be insufficient to establish an immune-mediated etiology. Immune-related toxicities may be severe, multisystem, and treatment limiting. Prompt evaluation should address both immune-mediated and alternative treatment-related, vascular, infectious, metabolic, epileptic, and malignant causes. Early multidisciplinary assessment is essential to reduce morbidity while avoiding unnecessary interruption of effective oncologic therapy. Continued reporting of such cases is needed to improve recognition, clarify risk factors, and guide management in rare malignancies such as vaginal cancer.

## Statement of Ethics

Ethical approval is not required for this study in accordance with local or national guidelines. Written informed consent was obtained from the patient for publication of the details of their medical case and any accompanying images.

## Conflict of Interest Statement

All authors declare no competing interests.

## Funding Sources

This study received no external funding.

## Author Contributions

C.E.S.: conceptualization, writing – original draft, and writing – review and editing. L.E.D.: writing – original draft and writing – review and editing. J.A.K. and L.B.: conceptualization, supervision, methodology, and writing – review and editing.

## Data Availability

No datasets were generated or analyzed for this study. The single patient-level data that support the findings of this study are not publicly available due to privacy reasons but are available from the corresponding author upon reasonable request.
